# Ultrabroadband Design for Linear Polarization Conversion and Asymmetric Transmission Crossing X- and K- Band

**DOI:** 10.1038/srep33826

**Published:** 2016-09-23

**Authors:** Linbo Zhang, Peiheng Zhou, Haiyan Chen, Haipeng Lu, Haiyan Xie, Li Zhang, En Li, Jianliang Xie, Longjiang Deng

**Affiliations:** 1 National Engineering Research Center of Electromagnetic Radiation Control Materials, State Key Laboratory of Electronic Thin Film and Integrated Devices, University of Electronic Science and Technology of China, Chengdu 610054, China; 2 School of Electronic Engineering, University of Electronic Science and Technology of China, Chengdu 610054, China

## Abstract

In this work, a high-efficiency and broadband reflective converter using ultrathin planar metamaterial (MM) composed of single-layered SRR is firstly realized. Numerical and experimental results demonstrate that the cross-polarization conversion reflectance above 0.84 is achieved from 8.6 to 18.6 GHz for linearly polarized (LP) incident waves under normal incidence. Subsequently, a multi-layered MM based on SRR enables a dramatic improvement of the recently demonstrated asymmetric transmission (AT) effect. Theoretical and measured results present that strong one-way transmission of two orthogonally polarized waves crossing C- and K- band has been observed. These two separated AT pass-bands have a function of selective polarization filter, which can be switched on/off by changing the polarization state of incident waves. The physical mechanisms are elucidated by taking advantage of electric fields and current distributions. Considering the broad bandwidth and the dual band, we believe that these two structures will be beneficial for designing polarization-controlled and selective transmission converter.

There is an increasing interest in using metal-dielectric structures to control the polarization and propagation states of electromagnetic (EM) waves^1^,^2^,^3^. In order to achieve such control it is necessary to create devices that have flexible manipulation of phase modulation and amplitude in reflection, transmission and absorption^4^,^5^,^6^,^7^. Metal-dielectric structures with strong birefringence and chirality have been demonstrated, with applications in quarter and half wave plates^8^,^9^,^10^, holograms^11^,^12^ and anomalous reflection^13^,^14^. Conventional polarization control devices based on optical gratings^15^ and dichroic crystals^16^ typically operate over narrow frequency bands, which may be broadened by stacking multiple structures or introducing a gradient in the helical pitch^17^,^18^. However, more bandwidth increases are usually achieved at the price of much bulkier devices, which are difficult to integrate within today’s miniaturized system.

Metamaterial (MM) – artificial EM structure with unconventional elements – has opened new routes towards the efficient manipulation of EM propagation due to its unusual properties^19^,^20^,^21^,^22^. Many basic MM structures, such as V-shaped antenna^1^, exhibit birefringence suitable for polarization conversion, which has been investigated in a number of theoretical and experimental works^23^,^24^,^25^,^26^,^27^. Broadband MM linear converters have been demonstrated in the short-wavelength infrared band by using high-refractive-index silicon cut-wire structure^28^. MMs-based polarimetric devices are particularly attractive in the microwave frequency range due to the lack of suitable natural materials for applications. Hao *et al*. demonstrated a reflective converter based on anisotropic MM, which could generate multiple resonance frequencies^29^. A MM converter -based electric-field-coupled resonator for such purpose was presented in refs 30 and 31. Meanwhile, linear converter in transmission mode is also interesting for many applications. Since the asymmetric transmission (AT) effect firstly observed by Fedotov *et al*.^32^, lots of sophisticated MM structures have been proposed^33^,^34^,^35^,^36^,^37^,^38^. Methods and designs have been proposed to enhance the magnitude of AT effect in the planar chiral MMs. In 2010, Menzel *et al*.^39^ reported a bi-layered chiral structure, achieving only magnitude of 0.25 for linearly polarized (LP) waves. Subsequently, Shi *et al*.^40^,^41^,^42^ proposed a kind of chiral MMs to achieve AT effect for LP waves. Despite its high polarization conversion efficiency, the above designs suffer from either very limited bandwidth or increased complexity. Recent years, some designs for broadband asymmetric transmission have been reported. In 2015, Cong *et al*.^43^ and Liu *et al*.^44^ presented L-shaped antenna in the teraherz range to manipulate EM waves. Cross-shaped MM was demonstrated by Wang *et al*.^45^,^46^, the AT bandwidth could be up to 33% of the central wavelength. Liu *et al*.^47^,^48^ proposed a multi-layered metallic structure, which can achieve a high magnitude of 0.94 and broadband AT effect for LP waves. In 2016, a cavity-based linear converter was showed by Wang *et al*.^49^, which exhibits very good performance with stable transmittance as 50%. Meanwhile, a three-layered sandwiched MM was proposed to achieve broadband AT effect in the near-infrared communication band^50^. The physical mechanisms of the polarization conversion and AT effect can be explicated by Lorentz-theory approach and Fabry-Perot like resonance model^3^,^47^,^48^,^51^,^52^. Thus, these intriguing features therefore make it possible to give us some new idea to design high magnitude and broadband converter.

In this work, we firstly present a high-efficiency and broadband reflective converter using ultrathin planar MM, which are capable of converting a LP wave into its orthogonal polarization. This device exhibits wideband property numerically as well as experimentally. The measured results show that the reflectance over 0.85 is achieved from 8.6 to 18.6 GHz for LP incident waves under normal incidence. In addition, a multi-layered MM based on SRR enables a dramatic improvement of the asymmetric transmission (AT) effect. Theoretical results show that the multi-layered MM can achieve cross-polarization with broad bandwidth and high efficiency for two orthogonal LP EM waves in two separated frequency regions, where LP waves can be mostly converted to its cross-polarization and then transmitted. The phenomenon can be functionalized as a dual-band polarization-selective filter by changing the polarization state of incident waves.

## Results and Discussion

### Unit cell design

**Figure 1:**
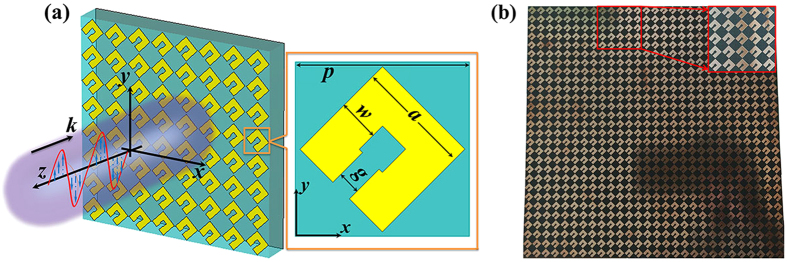
(**a**) Schematic of the reflective coverter. (**b**) Image of the sample with a unit cell period of *p *= 7.5, *a* = 5, *w* = 1.8, *g* = 1 and *t* = 2 (unit: mm).

A schematic of the converter is illustrated in Fig. 1(a). It is composed of a copper-patterned layer of split-ring resonator (SRR) and a continuous metal ground plane, with a dielectric layer of Teflon in between. The optimized unit cell is chosen as periodic dimensions of *p* = 7.5 mm in *x*-*y* plane, and the thickness of dielectric layer is *t* = 2 mm in the propagation of EM wave, *z* direction. The parameters of SRR are as follows: *a* = 5 mm, *w* = 1.8 mm and *g* = 1 mm.

The aforementioned geometrical parameters have been optimized by the commercial software CST Microwave Studio. In simulations, the conductivity of copper was *σ* = 5.8 × 10^7^ S/m, and the Teflon was simulated with a dielectric function of *ε* = 2.65 × (1 + 0.002*i*). The frequency domain solver was carried out with unit cell boundary condition in the *x*-*y* directions and the floquet ports in the *z*-direction to extract S parameters.

### Experimental results and characterization

**Figure 2:**
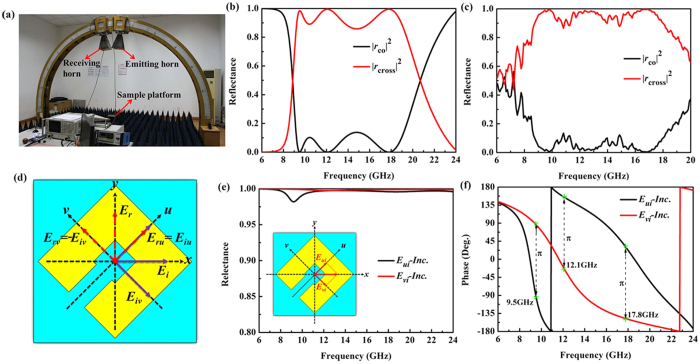
(**a**) Schematic of the polarization conversion measurement setup. (**b**) Simulated and (**c**) measured reflectance for co- and cross-polarized waves. (**d**) Schematic of the decomposition of linearly polarized incident and reflected waves. (**e**) Reflectance and (**f**) phase for a polarized wave along the *u*-and *v*-axes, respectively, demonstrating near-unity reflection and a π-phase shift under resonant frequencies.

Device fabrication was using the conventional printed circuit board propocess with the structural parameters same as the simulated model. The sample image is shown in Fig. 1(b), which has an oversize of 300 mm × 300 mm, containing 40 × 40 unit cells. To experimentally characterize the performance of the converter, the reflectance dependent on frequency was measured by the United States Naval Research Laboratory (NRL) arch method^53^ as shown in Fig. 2(a). An Agilent 8720ET vector network analyzer and two broadband horn antennas were connected by a coaxial cable. Two antennas were used to emit and receive EM waves. The reflectances |*r*_co_|^2^ and |*r*_cross_|^2^ can be measured by rotating the horn antenna in the arch structure, as illustrated in Fig. 2(c). Due to the limitations of experimental conditions, the measurement is conducted only in the range of 6–20 GHz. The experimental results demonstrate that |*r*_cross_|^2^ remains above 0.85 from 8.6 to 18.6 GHz, little difference with the simulations (9.2 to 19.2 GHz) shown in Fig. 2(b). In addition, the simulated reflectances |*r*_cross_|^2^ are 0.98, 0.99 and 0.99 at resonant frequencies of 9.5 GHz, 12.1 GHz and 17.8 GHz, respectively; |*r*_cross_|^2^ are 0.99, 0.99 and 1.0 at resonant frequencies of 9.8 GHz, 12.4 GHz and 16.9 GHz for experiments. The reason of the minor shift of the measured reflectances may be the limited precision of fabrication geometry as well as the dielectric board material whose actual dielectric constant is slightly different from the value used in simulations.

To better understand the response of the converter, the incident wave, which is LP along *x*-direction, can be decomposed into two perpendicular components, *u*- and *v*-axis, respectively (Fig. 2(d)). The numerical simulated reflection amplitude and phase for the converter illustrated with polarization along *u*- and *v*-axis are illustrated in Fig. 2(e,f). It can be seen that the reflection amplitudes in the two curves are nearly the same and close to unity while the relative phase retardation is roughly π at the three resonant frequencies of 9.5 GHz, 12.1 GHz and 17.8 GHz. Taking the first resonant frequency of 9.5 GHz, e.g., we decompose the electric field vector of the incident and reflected waves as depicted in Fig. 2(d). Incident electric field ***E***_***i***_ can be decomposed into two componenets ***E***_***iv***_ and ***E***_***iu***_. Since the reflection phase difference along *v* and *u* direction is π, the reflective electric field ***E***_***rv***_ (***E***_***ru***_) is equal to the incident electric field −***E***_***iv***_ (***E***_***iu***_). Then the total reflected field ***E***_***r***_ can be obtained and parallel to the *y*-axis, where the linear polarization conversion occurs, leading to a 90 degree polarization conversion. The same polarization conversion process applies for the other two resonant frequencies of 12.1 GHz and 17.8 GHz. We can understand the high conversion efficiency by modeling the field evolution upon multi-reflection process within the dielectric layer, where the consequence interference of polarization couplings results in constructive or destructive interference, respectively^3^,^27^,^54^,^55^. In addition, symmetric and antisymmetric modes supported by SRR are excited by the electric field configurations, which can be also explained the principle of our converter^1^,^56^.

Numerical simulations also show that the device maintains near-unity polarization conversion efficiency for variations of the dielectric spacer thickness and in the meantime, the high-frequency resonant peaks shift toward lower frequencies with increase of thickness *t* while the lowest resonant frequency hardly changes (Supporting Information Fig. S1). Similar situations are presented as variation of periodic unit *p*, for which the converter keeps conversion efficiency above 0.8 with bandwidth over 11.4 GHz for *p* ≤ 8.5 mm (Supporting Information Fig. S2). Meanwhile, variation of gap *g* and width *w* of SRRs could directively influence the positions of the highest and lowest resonant peaks, respectively (Supporting Information Figs S3 and S4).

In addition, the simulated and measured results show that this device is robust to variation of incident angle up to 30 degree for LP incident waves (Supporting Information Figs S5 and S6). However, the bandwidth of the converter is reduced when the incident angle is greater than 30 degree.

### Demonstration of A Dual-Band Polarization Filter

**Figure 3:**
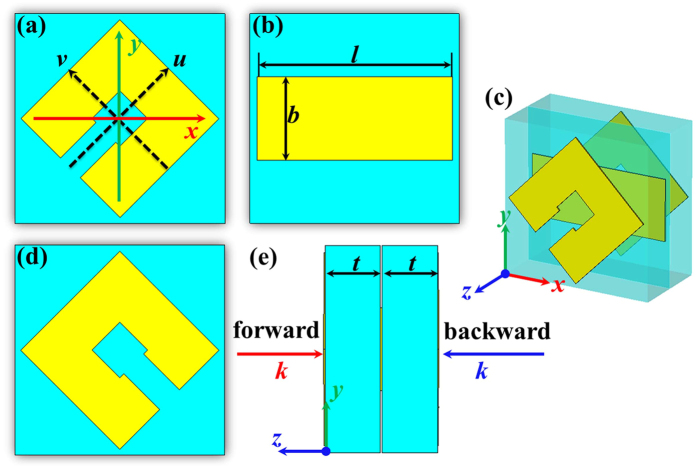
Schematic of the unit cell of the multi-layered linear converter. Parameters of the SRR array and the spacer are the same as that in Fig. 1. (**a**) Front view of the structure, (**b**) geometry of cut-wire in the middle layer, (**c**) perspective view of the structure, (**d**) bottom metallic layer, and (**e**) right side of structure. Dimension (mm): *p* = 7.5, *a* = 5, *w* = 1.8, *g* = 1, *l* = 7, *b* = 3 and *t *= 2.

Many applications require linear polarization conversion in transmission mode, for which we replace the metal ground plane in our design. By properly introducing a metal cut-wire, a multi-layered MM is proposed as shown in Fig. 3, which is composed of a cut-wire sandwiched by two layers of SRR. The front and back metallic layers are consisted of the same SRR, but with the split directions perpendicular to each other, while the middle layer is parallel to *x*-axis. Numerical results demonstrate that the multi-layered MM is nearly transparent to incident LP waves in two separated AT passbands, where a LP wave can be mostly converted to its cross-polarization and then transmitted, while the same LP wave cannot pass through the MM in the opposite direction.

In order to better understand the selective AT effect based on the proposed multi-layered MM, terms of complex transmission coefficients *T*_*ij*_^*d*^ in Jones matrix are presented, where *t*_*ij*_^*d*^ = |*T*_*ij*_^*d*^|. The subscripts *i* and *j* correspond to the polarization states of the transmitted and incident waves, which could be either *v* or *u* LP wave depicted in Fig. 3(a). The superscript *d* refers to the forward (*f*, along −*z* direction) or backward (*b*, along +*z* direction) wave propagations.

**Figure 4:**
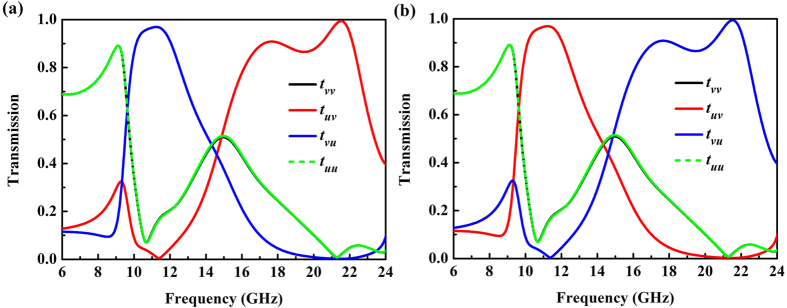
Transmission coefficients (absolute value) of linearly polarized waves in (**a**) forward (−*z*) direction and (**b**) backward (*z*) direction.

Figure 4 shows the simulated transmission coefficients in the multi-layered MM for the forward and backward propagating waves. The co-polarization transmission coefficient *t*_*vv*_ of *v*-polarized wave coincides well with *t*_*uu*_ of *u*-polarized wave under normal incidence. In contrast to co-polarization transmission, the cross-polarization transmission coefficient *t*_*uv*_ is extremely different from *t*_*vu*_ at the considered frequencies. These above two conditions ensure the presence of AT effect for LP waves^33^,^34^,^35^,^36^,^37^,^38^,^39^,^40^,^41^,^42^,^43^,^44^,^47^,^48^. Most interestedly, there are two separated passbands observed, demonstrating strong optical activity in the proposed MM, one for *v*-to-*u* polarization conversion and the other for *u*-to-*v* polarization conversion. In Fig. 4(a), the cross-polarization transmission coefficient *t*_*vu*_ reaches a maximum of 0.98 at around 11.2 GHz. *t*_*vu*_ above 0.8 can be achieved from 9.9 GHz to 12.5 GHz, and both the co-polarization transmission coefficients *t*_*vv*_ and *t*_*uu*_ are below 0.3. In this pass band, the *v*-polarized incident wave is mostly transmitted to *u*-polarized wave while the *u*-polarized incident wave is mostly blocked through the MM. Meanwhile, another obvious broad band for *t*_*uv*_ above 0.8 can be observed from 16.1 GHz to 22.5 GHz. The cross-polarization transmission coefficient *t*_*uv*_ reaches 0.91 and 0.99 at two resonant frequencies of 17.5 GHz and 21.5 GHz, respectively. Similarly, in this broad pass band, a *v*-polarized incident wave is mostly transmitted to *u*-polarized wave, while the *u*-polarized incident wave cannot pass through the MM. Otherwise, *t*_*uv*_ and *t*_*vu*_ interchange with each other when the propagation direction is reversed as depicted in Fig. 4(b).


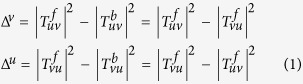


**Figure 5:**
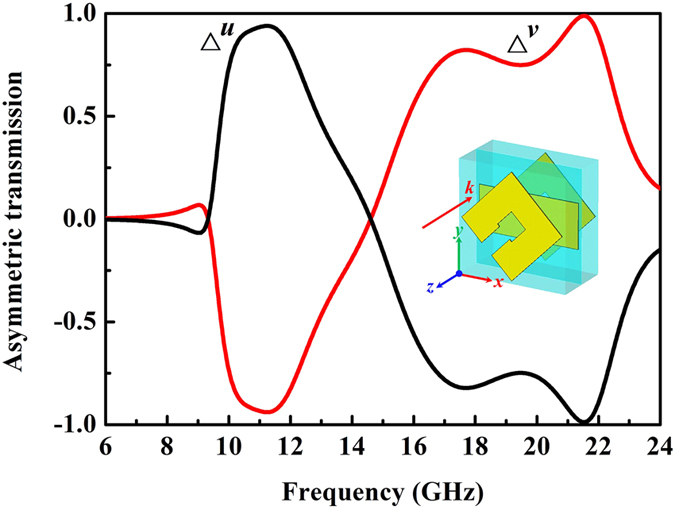
Calculated asymmetric transmission parameter Δ. Black and red solid curves correspond, respenctively, to asymmetric transmissions of *v* and *u* linearly polarized waves in the forward (−*z*) propagation direction.

As depicted in Fig. 4, *t*_*vv*_ is exactly equal to *t*_*uu*_, which ensures zero AT effect of circular polarization waves but only for LP waves by this MM^32^,^33^,^34^,^35^,^36^. The AT parameter Δ can be calculated by Eq. (1), which is characterized as the difference between the transmissions of two opposite propagation directions^34^. Figure 5 presents the calculated AT parameter Δ for forward *u*- and *v*-polarized incident waves according to Eq. (1). It is observed that AT parameters are 0.94/−0.94, −0.82/0.82 and −0.99/0.99 for Δ^*u*^/Δ^*v*^ at resonant frequencies of 11.2 GHz, 17.5 GHz and 21.5 GHz, respectively. Both Δ^*u*^ and Δ^*v*^ depict two opposite passbands over 0.75, respectively, from 10.0 to 12.2 GHz and from 16.8 to 22.3 GHz, which implys that a *u*/*v*-polarized wave in the forward direction is mostly allowed/forbidden in the former pass band, and forbiddened/allowed in the latter pass band. This interesting phenomenon demonstrates that the proposed MM can be used as a selective dual-band polarization filter. In addition, it is observed that the multi-layered MM maintains this broadband selective transmission property for incident angle up to 30 degree (Supporting Information Fig. S7).

**Figure 6:**
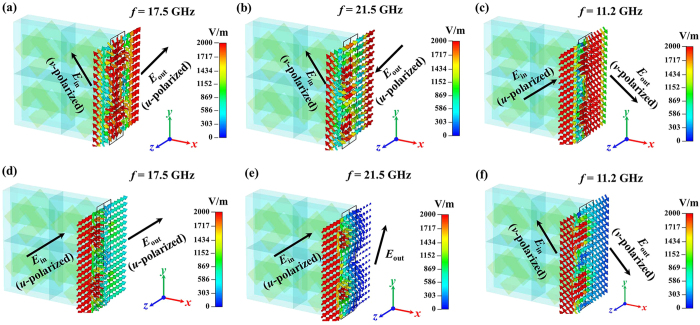
The electric field configurations in the multi-layered MM at resonant frequencies of 17.5 GHz, 21.5 GHz and 11.2 GHz for (**a**,**b**,**f**) a *v*-polarized wave, and (**c**–**e**) a *u*-polarized wave propagating along the forward (−*z*) direction.

To understand the physical insight of the LP waves passed through the proposed MM, electric field configurations for incident waves along forward direction at resonant frequencies of 11.2 GHz, 17.5 GHz and 21.5 GHz are presented in Fig. 6. For the proposed MM, the electric fields depicted of *v*-polarized wave shown in Fig. 6(a,b) and *u*-polarized wave shown in 6(c) have been converted by 90°, which is clear that the *v*/*u*-polarized incident wave is transformed into an *u*/*v*-polarized output wave at the resonant frequencies. It is nearly transparent for a *u*/*v*-polarized wave shown in Fig. 6(d,f) but reflects most of the *u*/*v*-polarized wave as depicted in Fig. 6(e,f). By observation of the electric field magnitudes as shown in Fig. 6(d–f), we can see that reflectances *t*_*uu*_ is about 0.3 and 0.05 at frequencies of 17.5 GHz and 21.5 GHz, respectively; *t*_*uv*_ is around 0.2 at frequency of 11.2 GHz. These results coincide with the analysis results in Fig. 4.

**Figure 7:**
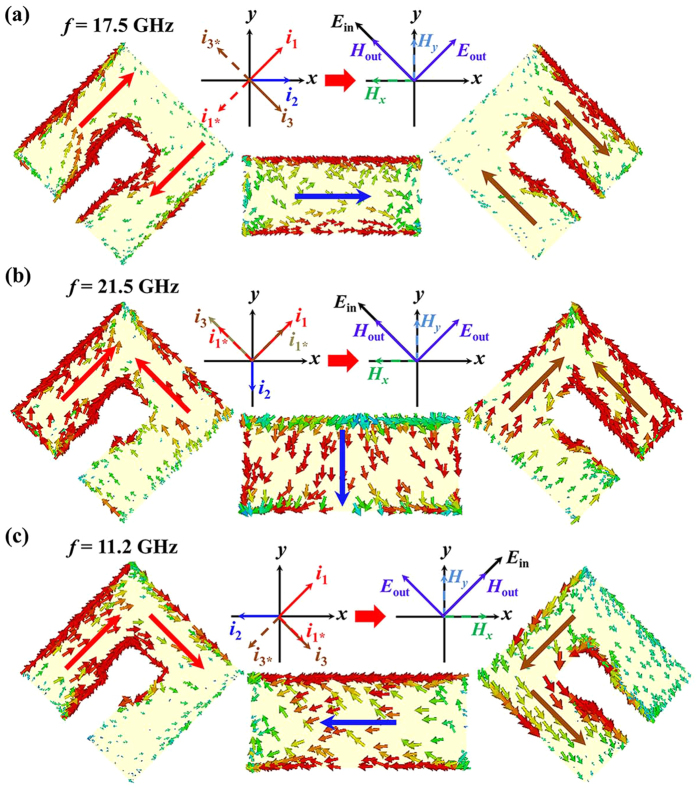
Surface current distributions of the single-layered pattern for forward propagating (**a**,**b**) *v*- and (**c**) *u*-polarized waves at resonant frequencies of 17.5 GHz, 21.5 GHz and 11.2 GHz, respectively. The schematic current distribution is represented by colors on the metallic patterns (red for front SRR, blue for cut-wire, and brown for back SRR). The direction of current flow is indicated by arrows.

In order to understand the physical origin of the two passbands of AT effect, surface current distributions of the proposed MM are presented at the resonant frequencies of 17.5 GHz, 21.5 GHz and 11.2 GHz, respectively. In Fig. 7, the red, blue and brown solid arrows represent the current direction of top SRR layer, middle cut-wire layer and bottom SRR layer, respectively. As we know, the coupling effect between electric and magnetic fields plays an important role in a chiral or anisotropic MM^57^,^58^,^59^. It is observed from Fig. 7(a) that the induced *x* component ***H***_***x***_ and *y* component ***H***_***y***_ generated by the anti-symmetric and symmetric currents decomposed in *x* and *y* direction. Then the total magnetic field ***H***_***out***_ parallel to incident electric filed***E***_***in***_ can be obtained. The cross-coupling between ***E***_***in***_ and ***H***_***out***_ leads to a cross-polarization with a *v*-to-*u* polarization conversion. Thus, most energy of the *v*-polarized incident wave are transformed with a 90° rotation at resonant frequency of 17.5 GHz. Similarly, the transmitted features with polarization conversion occur at the other two resonant frequencies of 21.5 GHz and 11.2 GHz as shown in Fig. 7(b,c).

**Figure 8:**
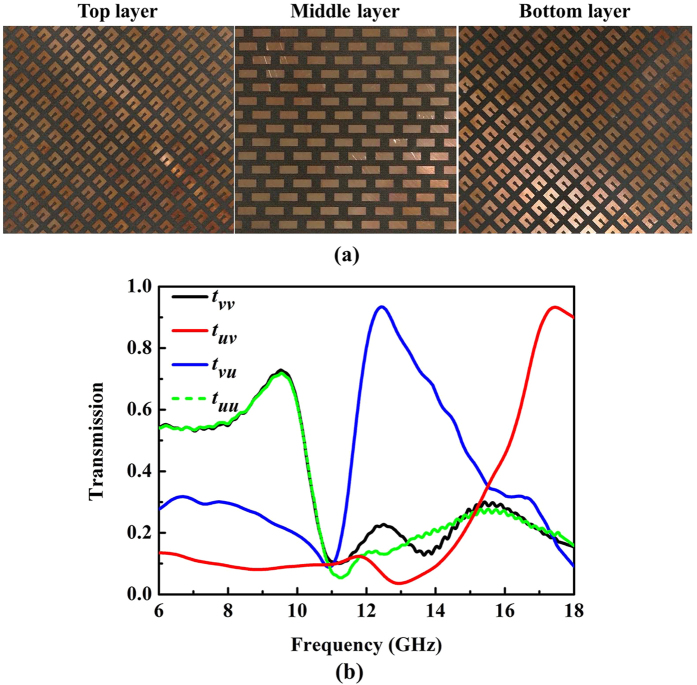
Photographs of the top, middle and bottom of the experimental sample, respectively. (**b**) Measured transmission coefficients *t*_*vv*_, *t*_*uv*_, *t*_*vu*_ and *t*_*uu*_ for forward propagating waves under normal incidence.

To validate the AT effect and dual-band polarization of the multi-layered structure, a 300 mm × 300 mm sample of the proposed structure is fabricated and measured, as shown in Fig. 8. Due to the limitations of experimental conditions, the measurement is conducted only in the range of 6–18 GHz. As depicted in Figure 8(b), we can see that the co-polarization transmission coefficient *t*_*vv*_ of *v*-polarized wave nearly coincides well with *t*_*uu*_ of *u*-polarized wave. Meanwhile, *t*_*uv*_ is extremely different from the *t*_*vu*_ at the considered frequencies. These phenomena verify the presence of AT effect for the proposed structure. The polarization conversion bandwidth of *t*_*uv*_ over 0.75 can be achieved from 11.9 GHz to 13.4 GHz; transmission coefficient *t*_*uv*_ is 0.93 at resonant frequency of 12.4 GHz. *t*_*vu*_ above 0.75 can be achieved from 16.7 GHz to 18.0 GHz; and *t*_*vu*_ reaches a maximum of 0.93 at resonant frequency of 17.4 GHz. However, we can see a discrepancy of the cross-polarization transmission coefficient *t*_*uv*_ and *t*_*uv*_ between the simulated and measured result. This is due to the small signal to noise ratio (SNR) for the test system in the process of calibration. Even though the discrepancy, AT effect and separated dual-band polarization conversion can still be realized.

## Conclusion

In summary, we have firstly demonstrated a reflective converter composed of a single-layered SRR both numerically and experimentally. The proposed converter can accomplish high efficiency and broad bandwidth for LP waves. Experimental results show that the cross-polarizaton conversion reflectance above 0.84 is achieved from 8.6 to 18.6 GHz for LP incident waves under normal incidence, and above 0.8 from 9.2 to 18.5 GHz for incident angle of 30 degree. Subsequently, a multi-layered MM based on SRR enables a dramatic improvement of the recently demonstrated asymmetric transmission (AT) effect, different from the properties in the previous papers^34^,^35^,^36^,^37^,^38^,^39^,^40^,^41^,^42^,^47^,^48^. It is found that the cross-polarization transmission coefficient over 0.8 of the proposed multi-layered MM can be achieved from 9.9 to 12.5 GHz for the *u*-polarized wave and from 16.1 to 22.5 GHz for the *v*-polarized wave in the two pass-bands, respectively. The broadband property can be maintained for incident angle up to 30 degree. These two separated AT pass-bands have a function of selective polarization filter, which can be switched on/off by changing the polarization state of incident waves. The physical mechanisms are elucidated by taking advantage of electric fields and current distribution. With the properties of broad bandwidth and high-efficiency, we believe that these two structures will be beneficial for designing polarization-controlled and selective transmission converter.

## Methods

Finite element method modeling was performed using commercial software CST Microwave Studio. The frequency domain solver was carried out and a frequency sweep of 6–24 GHz used to produce the results shown in this paper.

To obtain the polarization conversion characteristic, both co-polarized and cross-polarized signals were measured. For the reflective converter, the sample was placed on the test platform in the experiment. The distance between antennas and sample was chosen far enough to avoid near field effect. Two standard linearly polarized horn antennas served as transmitter and receiver, respectively. An Agilent 8720ET vector network analyzer and the horn antennas were connected by a coaxial cable. Through rotating the receiving antenna by 90 degree, the horn antennas can be reconfigured between *x* and *y* polarization modes, so that we can obtain both the co- and cross-polarized reflectance. The separation angle between the two antennas is set to be less than 5 degree, which can be considered as the case of the normal incidence. For oblique incidence. the transmitting and receiving horn antennas were both moved along the arch trace structure to measure the reflectance.

## Additional Information

**How to cite this article**: Zhang, L. *et al*. Ultrabroadband Design for Linear Polarization Conversion and Asymmetric Transmission Crossing X- and K- Band. *Sci. Rep*. **6**, 33826; doi: 10.1038/srep33826 (2016).

## Supplementary Material

Supplementary Information
