# Supplementary material for: Ultrabroadband Design for Linear Polarization Conversion and Asymmetric Transmission Crossing X- and K- Band
Source: Sci Rep. 2016 Sep 23;6:33826. doi: 10.1038/srep33826 (PMC5034340; doi:10.1038/srep33826)
Supplement: Supplementary Information [file srep33826-s1.pdf]

Ultrabroadband Design for Linear Polarization Conversion and Asymmetric Transmission Crossing X- and K- Band

Linbo Zhang[1], Peiheng Zhou[1*], Haiyan Chen[1], Haipeng Lu[1], Haiyan Xie[1], Li Zhang[1], En Li[2], Jianliang Xie[1] and Longjiang Deng[1]

[1]National Engineering Research Center of Electromagnetic Radiation Control Materials, State Key Laboratory of Electronic Thin Film and Integrated Devices, University of Electronic Science and Technology of China, Chengdu 610054, China.

[2]School of Electronic Engineering, University of Electronic Science and Technology of China, Chengdu 610054, China.

*Corresponding author: Peiheng Zhou; zhouph1981@163.com

Supplementary Information

1. Simulations of the reflective converter

a. Device performance with varying Teflon thickness *t*

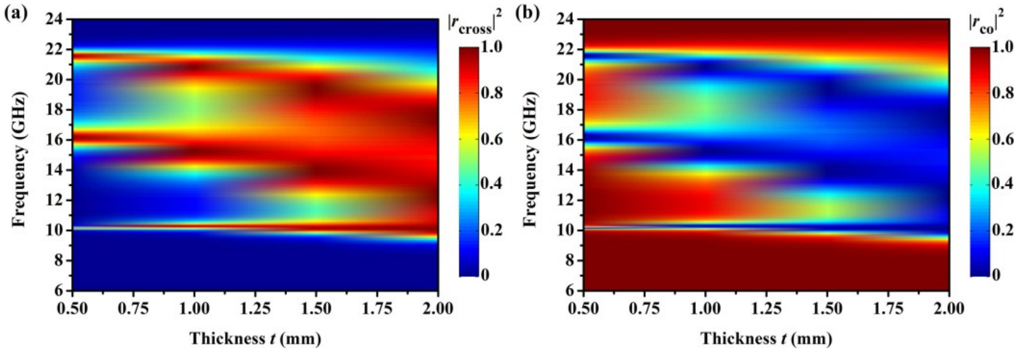

Fig. S1. (a) Cross-polarzation reflectance $|r_{\mathrm{cross}}|^2$ and (b) co-polarization reflectance $|r_{\mathrm{co}}|^2$ of the converter with varying thickness t. Dimension (mm): p = 7.5, a = 5, w = 1.8, g = 1 and t.

The optimized Teflon thickness is dertermined through a though parameter sweep using CST simulation. Fig. S1(a) and S1(b) depict the simulated reflectances $|r_{\mathrm{co}}|^2$ and $|r_{\mathrm{cross}}|^2$ for different thickness t. As can be observed, the polarization conversion efficiency at the three resonant frequencies is near-unity for thickness ranging from 0.5 mm to 2.0 mm. When thickness t is 2.0 mm, the bandwidth of reflectance $|r_{\mathrm{cross}}|^2$ over 0.8 can be achieved from 9.2 to 19.2GHz. In addition, we can see that the thickness only affect the latter two high resonant frequencies, while the lowest-resonance frequency hardly changes.

b. Device performance with varying periodic unit *p*

The effect of variation of p on polarization conversion can be seen in Fig. S2. We can observe that the second resonant peak shits toward high frequency with increasing p. However, the other two resonant peaks cannot be suffered from the influence of different p. And when $p \leq 8.5$mm, the polarization conversion bandwidth of $|r_{cross}|^2 > 0.8$ can be achieved from 9.6GHz to 20.0GHz.

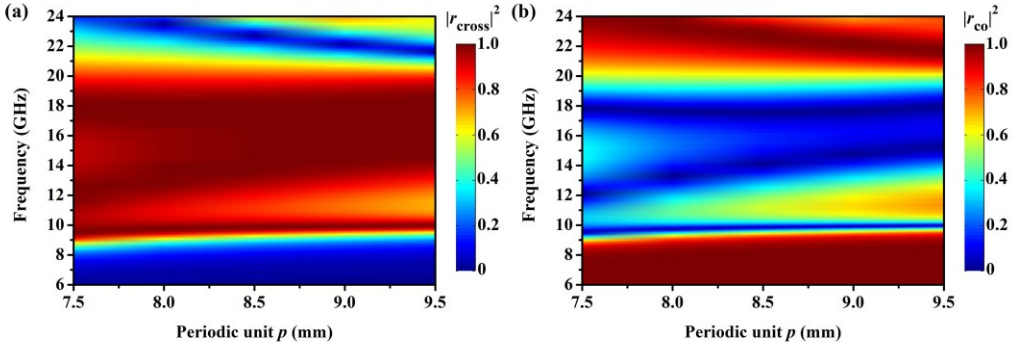

Fig. S2. (a) Cross-polarzation reflectance $|r_{cross}|^2$ and (b) co-polarization reflectance $|r_{co}|^2$ of the converter with varying periodic unit p. Dimension (mm): p, $a = 5$, $g = 1$, $t = 2$ and $w = 1.8$.

c. Device performance with varying gap g of SRRs

The similar phenomenon can be seen when varying the gap g of SRRs, as shown in Fig. S3. The position of the third resonant peak is determined by g. With increasing of g, the resonant peak shifts toward high frequency. And the polarization conversion of $|r_{cross}|^2$ over 0.8 can be achieved from 9.2GHz to 18.5GHz.

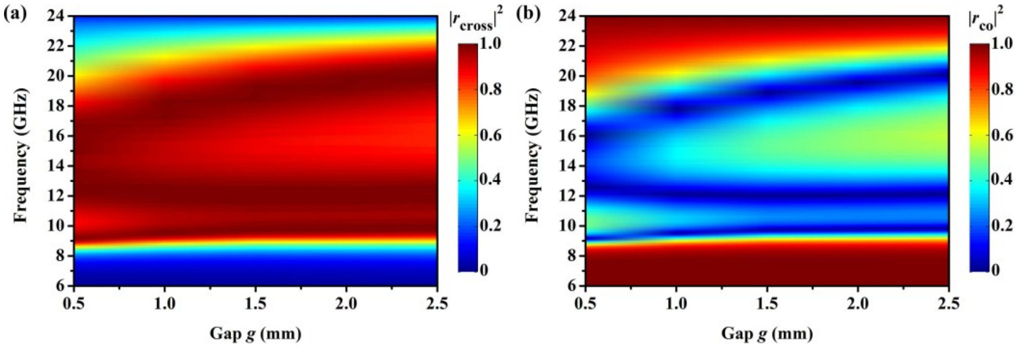

Fig. S3. (a) Cross-polarzation reflectance $|r_{cross}|^2$ and (b) co-polarization reflectance $|r_{co}|^2$ of the converter with varying gap g of SRRs. Dimension (mm): $p = 7.5$, $a = 5$, g, $t = 2$ and $w = 1.8$.

d. Device performance with varying width w of SRRs

In this section, as shown in Fig. S4, we can observe that it is robust to variation of width w for the latter two resonant frequencies. However, with decrease of w, the first resonant peak shifts toward lower frequency. Even so,

the device can maintain a broad bandwidth of $|r_{\mathrm{cross}}|^2$ over 0.9 from 11.1 GHz to 18.7 GHz when the width is 1.8mm $\geq w \geq$ 0.9. Based on the analysis of the structural parameters, it is obtained that the position of the three resonant peaks are determined by w, p and g, respectively.

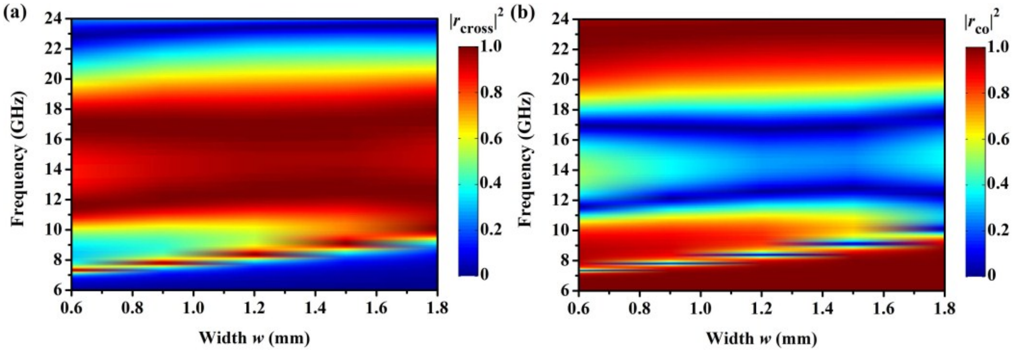

Fig. S4. (a) Cross-polarzation reflectance $|r_{\mathrm{cross}}|^2$ and (b) co-polarization reflectance $|r_{\mathrm{co}}|^2$ of the converter with varying width w. Dimension (mm): p = 7.5, a = 5, g = 1, t = 2 and w.

e. Device performance with changing incident angle θ

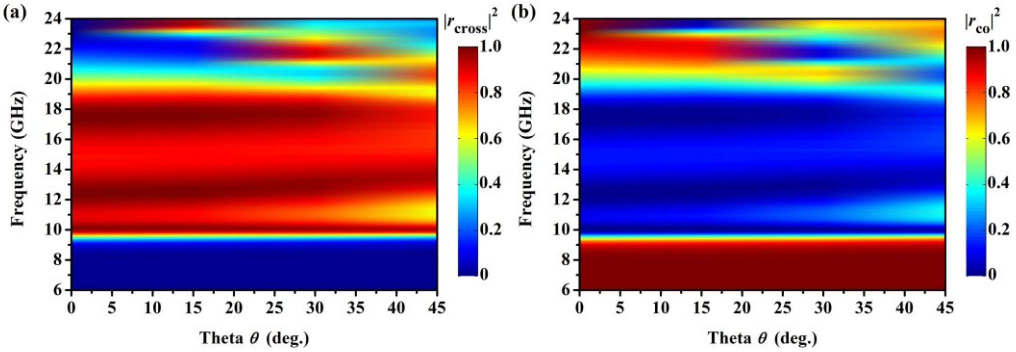

Fig. S5. (a) Cross-polarzation reflectance $|r_{\mathrm{cross}}|^2$ and (b) co-polarization reflectance $|r_{\mathrm{co}}|^2$ of the converter as a function of incident angle θ. Dimension (mm): p = 7.5, a = 5, w = 1.8, g = 1 and t = 2.

Here, we provide the oblique incident response of the proposed converter for y-polarized wave, as shown in Fig. S5. From the result, we observe an almost angle-dependent performance up to 45 degree for the resonant peaks. Such insensitivity to incident angle provides convenience in practical applications. Also, the bandwidth is robust to variation of incident angle up to 30 degree. However, it decreases resulting from a further reduction of $|r_{\mathrm{cross}}|^2$ between the first and second resonant frequencies with incident angle greater than 30 degree. Fig. S6 gives the simulated and measured results when incident angle is above 30 degree. Due to the limitations of experimental conditions, the measurement is conducted only in the range of 6-20 GHz. The measured results show that the device is robust to the variation of incident angle up to 30 degree. And the bandwidth of $|r_{\mathrm{cross}}|^2$ over 0.8 can be achieved from 9.2 to 18.5 GHz with inciden angle of 30 degree.

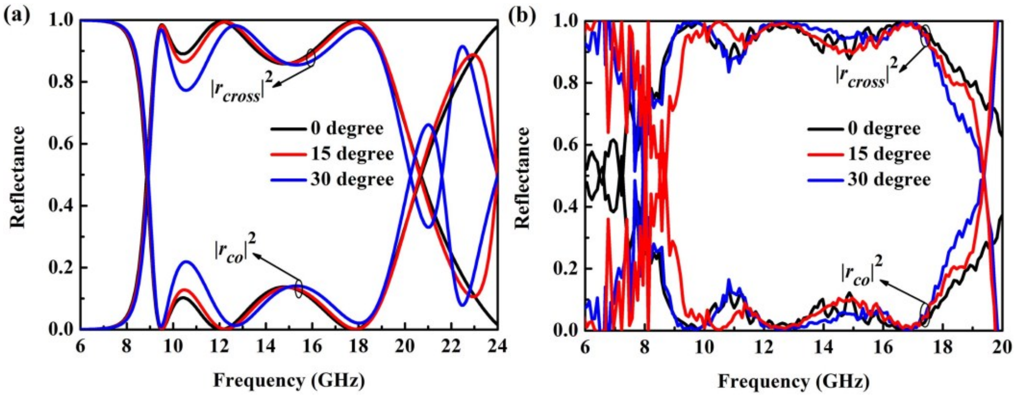

Fig. S6. Simulated and measured data of reflectance $|r_{\mathrm{cross}}|^2$ and $|r_{\mathrm{co}}|^2$ of the converter as incident angle θ is 30 degree. Dimension (mm): p = 7.5, a = 5, w = 1.8, g = 1 and t = 2.

2. Property of the multi-layered MM

a. Device performance with varying incident angle

The angle-dependent response of the multi-layered MM for *u*- and *v*-polarized waves is shown in Fig. S7. From the results, the broadband properties can be sustained as increase of incident angle up to 30 degree. For a *u*-polarized wave propagating along forward direction as shown in Fig. S7(a), the bandwidth of cross-polarization transmission t_{vu} over 0.8 can be achieved from 9.9 GHz to 12.0 GHz when increasing incident angle up to 45 degree. And for a *v*-polarized wave (Fig. S7(b)), the bandwidth of t_{uv} over 0.8 can be obtained from 15.6 GHz to 21.2 GHz with incident angle up to 30 degree. The insensitivity to incident angle ≤ 30 degree of these two AT pass-bands can be functionalized as a selective polarization transmission device.

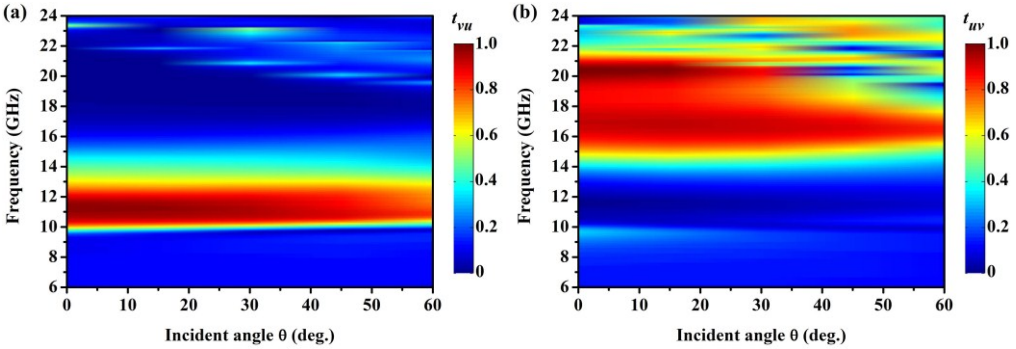

Fig. S7. Transmission coefficients (absolute value) (a) t_{vu} and (b) t_{uv} of linearly polarized wave in forward (-*z*) direction as a function of incident angle. Dimension (mm): p = 7.5, a = 5, w = 1.8, g = 1, l = 7, b = 3 and t = 2.
